# Tupaia small RNAs provide insights into function and evolution of RNAi-based transposon defense in mammals

**DOI:** 10.1261/rna.048603.114

**Published:** 2015-05

**Authors:** David Rosenkranz, Stefanie Rudloff, Katharina Bastuck, René F. Ketting, Hans Zischler

**Affiliations:** 1 Institute of Anthropology, Johannes Gutenberg-University, Mainz, Rheinland-Pfalz 55128, Germany; 2 Institute of Molecular Biology, IMB. Mainz, Rheinland-Pfalz 55128, Germany

**Keywords:** RNA interference, piRNA, siRNA, evolution, transposon defense

## Abstract

Argonaute proteins comprising Piwi-like and Argonaute-like proteins and their guiding small RNAs combat mobile DNA on the transcriptional and post-transcriptional level. While Piwi-like proteins and associated piRNAs are generally restricted to the germline, Argonaute-like proteins and siRNAs have been linked with transposon control in the germline as well as in the soma. Intriguingly, evolution has realized distinct Argonaute subfunctionalization patterns in different species but our knowledge about mammalian RNA interference pathways relies mainly on findings from the mouse model. However, mice differ from other mammals by absence of functional Piwil3 and expression of an oocyte-specific Dicer isoform. Thus, studies beyond the mouse model are required for a thorough understanding of function and evolution of mammalian RNA interference pathways. We high-throughput sequenced small RNAs from the male *Tupaia belangeri* germline, which represents a close outgroup to primates, hence phylogenetically links mice with humans. We identified transposon-derived piRNAs as well as siRNAs clearly contrasting the separation of piRNA- and siRNA-pathways into male and female germline as seen in mice. Genome-wide analysis of tree shrew transposons reveal that putative siRNAs map to transposon sites that form foldback secondary structures thus representing suitable Dicer substrates. In contrast piRNAs target transposon sites that remain accessible. With this we provide a basic mechanistic explanation how secondary structure of transposon transcripts influences piRNA- and siRNA-pathway utilization. Finally, our analyses of tree shrew piRNA clusters indicate A-Myb and the testis-expressed transcription factor RFX4 to be involved in the transcriptional regulation of mammalian piRNA clusters.

## INTRODUCTION

The fundamental task of an animal's germline is passing genomic information unscathed from one generation to another. In virtually all animal species the germline-expressed Piwi proteins and Piwi-interacting (pi-) RNAs protect genomes from a persistent bombardment of transposable elements (TEs) during epigenetic reprogramming (Malone and Hannon 2009; Thomson and Lin 2009). The Piwi/piRNA machinery acts on both the transcriptional as well as the post-transcriptional level. piRNAs that are encoded in large genomic clusters guide Piwi effector proteins to their targets based on sequence complementarity. In a nuclear context, this can result in chromatin modification, DNA methylation and repressed transcription of TEs (Carmell et al. 2007; Kuramochi-Miyaga et al. 2008; Sienski et al. 2012; Le Tomas et al. 2013; Rozhkov et al. 2013). Cytoplasmic Piwi activity leads to endonucleolytic cleavage of TE transcripts (7). In the latter case, TE transcripts can be processed into a second class of piRNAs that target transcripts of piRNA clusters, thus initiating a so-called ping-pong amplification loop (Brennecke et al. 2007; Gunawardane et al. 2007).

Besides piRNAs, another class of small noncoding (snc-) RNAs termed short interfering (si-) RNAs was found to play a crucial role in transposon defense in a variety of organisms (Ketting et al. 1999; Tabara et al. 1999; Sijen et al. 2003; Batista et al. 2008; Chung et al. 2008; Czech et al. 2008; Das et al. 2008; Ghildiyal et al. 2008; Hayashi et al. 2008; Tam et al. 2008; Flemr et al. 2013). In contrast to piRNAs, siRNAs arise from Dicer dependent processing of double-stranded (ds-) RNA precursors (Slotkin and Martienssen 2007). Regarding the interplay of the piRNA- and the siRNA-pathway, the available data reveal different Argonaute sub-functionalization patterns in different species. While TE-derived endogenous (endo-) siRNAs in *Drosophila melanogaster* are supposed to represent the somatic piRNA counterpart (Ghildiyal et al. 2008), siRNAs in *Caenorhabditis elegans* suppress DNA transposons also in the germline in a Piwi/piRNA dependent manner (Batista et al. 2008; Das et al. 2008). In mice, siRNAs and an oocyte-specific Dicer isoform are essential for female fertility (Flemr et al. 2013).

The factors that determine which pathway is dominant remain elusive, especially regarding systems where TE-related siRNAs and piRNAs are expressed at the same time and place. The situation becomes further complicated by the fact that for reasons of lineage-specific peculiarities of the RNAi machinery insights from the mouse model are not necessarily applicable to other mammals. The variability observed in the species studied thus far begs for analyses of sncRNA pathways in germ cells of additional species, including species that are less accessible to experimental manipulation. Only a comparative extension of our view on sncRNA pathways will allow us to grasp the essence of these extremely important silencing pathways.

**FIGURE 1.**
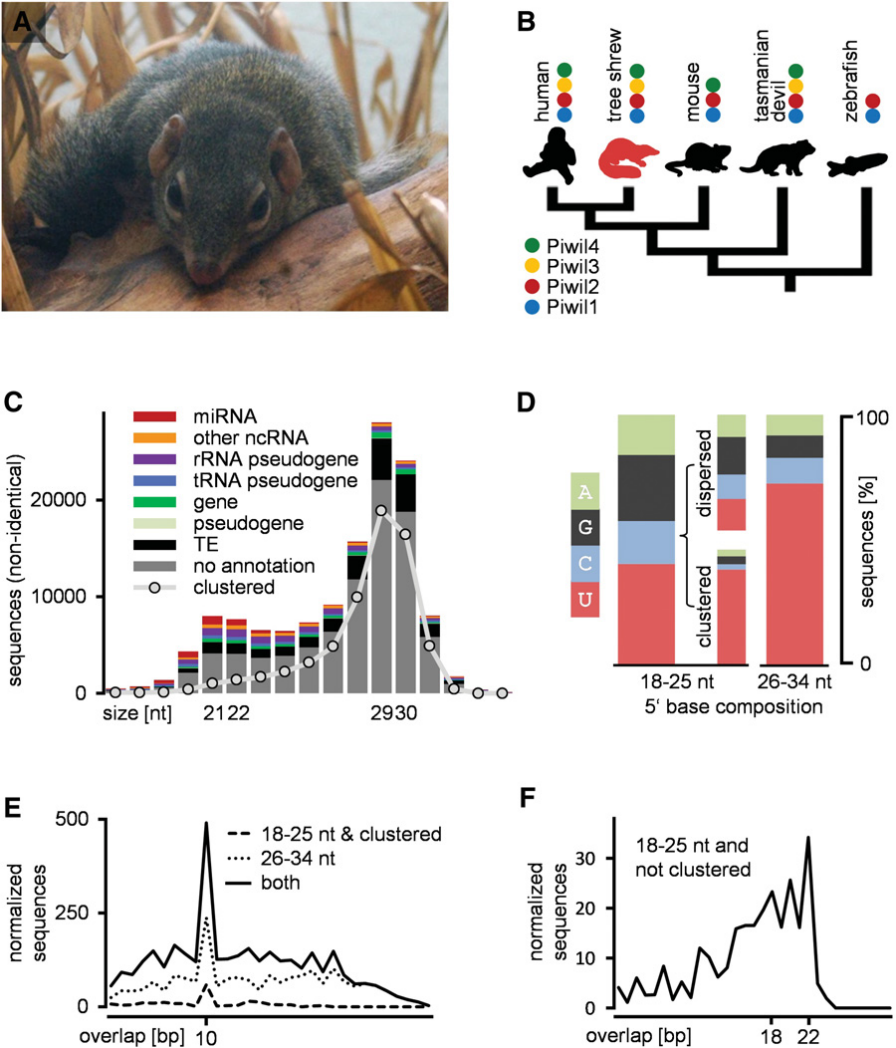
Analyses of testis-expressed small RNAs from the *Northern Tupaia*. (*A*) The *Northern Tupaia* (*Tupaia belangeri*, Zoo Frankfurt). (*B*) Phylogenetic relationship of the tupaia and presence/absence patterns of Piwi proteins in selected species. (*C*) Length profile and sequence annotation. (*D*) 5′ base composition of different small RNA fractions. (*E*) Ping-pong signature: preference for a 10 bp overlap of different sRNA fractions. (*F*) Interspersed 18–25 nt sequences do not show a ping-pong signature but tend to overlap with 18–22 nt. (*E*,*F*) Sequence counts refer to nonidentical sequences normalized by the number of genomic hits produced by each sequence.

We have therefore analyzed the sncRNA repertoire of the northern tree shrew (*Tupaia belangeri*, order: Scandentia, Fig. 1A, in the following abbreviated to tupaia). This close relative to primates has often been used as a model for research into human disease, learning, aging, social stress, and depression (Fan et al. 2013). Furthermore, the tupaia represents an important realization of genome evolution during Euarchontan divergence. Its TE landscape is not dominated by the Alu sequence that burst in primate evolution but shows a remarkable amount of tupaia-specific tRNA-derived Short Interspersed Nuclear Elements (Tu-SINEs2/3) with 21% of the whole genome being constituted of these elements. As for most mammals, including human, but in contrast to mouse and rat, its genome encodes four functional Piwi paralogs, which makes it an interesting animal to study with respect to the human Piwi/piRNA system (Fig. 1B).

Here we show that not only the Piwi/piRNA system, but also Dicer dependent processing of TE transcripts represents a major source of TE-related sncRNAs. The distribution of piRNA and siRNA target sites correlates well with the secondary structure of TE transcripts and corresponds to the mechanistic features of piRNA- and siRNA biogenesis. We outline a model in which TE transcripts that are barely accessible for piRNAs due to forming foldback secondary structures, thus potentially bypassing the piRNA-pathway, are tackled and processed by Dicer. Moreover, based on sequence analyses of tupaia piRNA clusters we introduce the testis-expressed transcription factor RFX4 as a promising candidate for transcriptional regulation of mammalian piRNA clusters.

## RESULTS

### Identification of different small RNA (sRNA) classes in the tupaia male germline

In total 130,390 nonidentical sRNA sequences representing ∼12.4 million sequence reads were successfully mapped to the tupaia genome resulting in ∼10.5 million perfect genomic hits. In total, 7344 sequences produced perfect matches to known noncoding (nc-) RNAs (Supplemental Table 1) and were annotated as miRNAs (313) or putative fragments of miRNA-precursors (3546), snoRNAs (1258), snRNAs (770), miscRNAs (696), rRNAs (472), or tRNAs (289). The bimodal length profile of the remaining 123,046 nonidentical sequences revealed discrete peaks at 22/23 nt and 29/30 nt, respectively (Fig. 1C). As these sequences do not correspond to other known ncRNA and in consistency with the observed length distribution, we presumed the two RNA fractions to comprise endo-siRNAs (18–25 nt) and piRNAs (26–34 nt), respectively, and analyzed the data set accordingly.

### Identification of piRNAs

We first checked for typical piRNA features like the 1U bias that results from 5′ Uridine recognition of Piwi proteins (Cora et al. 2014). We found that the 26–34 nt fraction, but not the 18–25 nt fraction exhibits a strong bias for Uridine at position 1 (Fig. 1D). Based on mapped sRNA reads we annotated genomic piRNA clusters and found that 64% of the sequences from the 26–34 nt fraction fell into one of 47 genomic clusters with a total size of 963,212 bp (Supplemental Table 2). The predicted clusters display typical piRNA cluster features previously described in various species (Aravin et al. 2007) and will be discussed in more detail below. Surprisingly, 23% of the sequences from the 18–25 nt fraction, thus sequences below the canonical piRNA length, also mapped to piRNA clusters (Fig. 1C). To check whether these sequences exhibit further piRNA features we compared clustered 18–25 nt sequences and dispersed 18–25 nt sequences with respect to the amount of 5′ U. In contrast to the dispersed sequences, the clustered sequences exhibit a strong preference for 5′ U as do the sequences from the 26–34 nt fraction (Fig. 1D). Therefore, it is likely that sequences <26 nt and mapping to piRNA clusters, and sequences ≥26 nt represent piRNAs. Since piRNAs preferentially overlap with 10 bp of their 5′ ends (ping-pong signature), we analyzed the 5′ overlap of these sequences. Strikingly, each fraction apart displays the typical ping-pong signature representing a hallmark of secondary piRNA biogenesis (Fig. 1E). In addition, the number of ping-pong pairs increased when we pooled the two fractions, illustrating that ping-pong pairs can also be formed between the differentially sized fractions.

**FIGURE 2.**
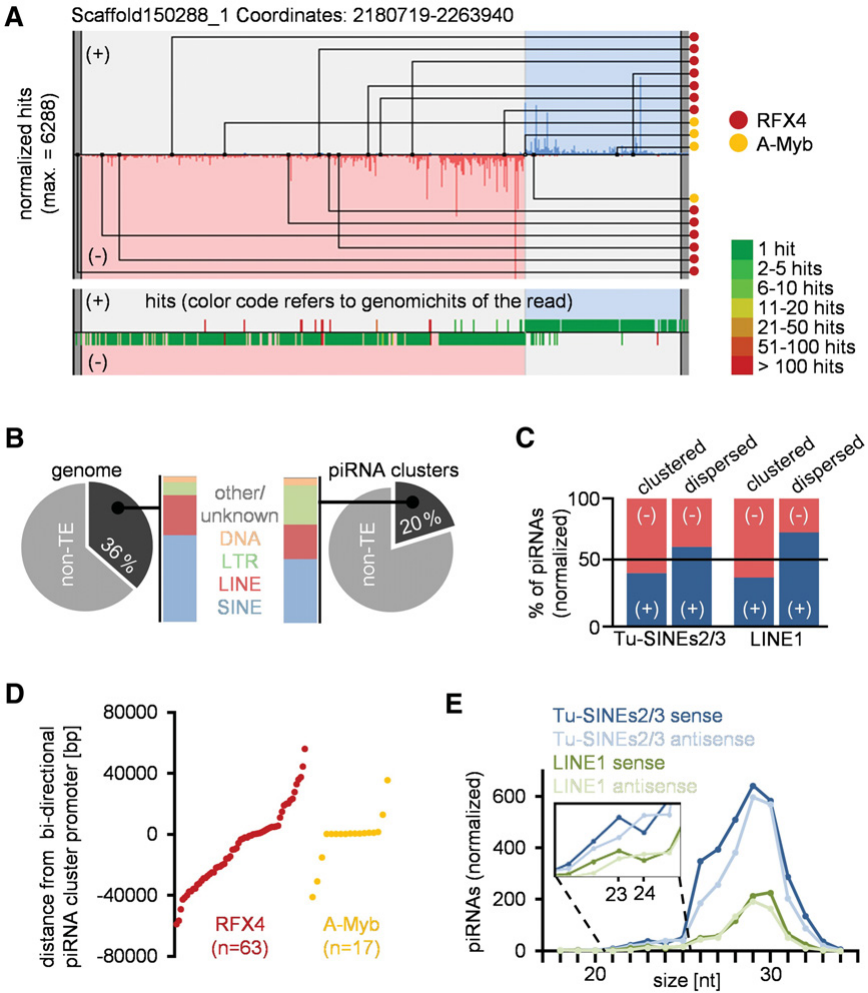
Characteristics of tupaia piRNA clusters. (*A*) Example of a bidirectional piRNA cluster (proTRAC output, modified). Binding sites for A-MYB and RFX4 transcription factors are indicated (*top*). Most sequence reads map to unique loci (*bottom*). (*B*) TE content and composition of piRNA clusters compared with the whole genome. (*C*) TE-related piRNAs in piRNA clusters are biased toward antisense orientation. Dispersed TE-related sequences are biased toward sense orientation. (*D*) Location of predicted RFX4 and A-Myb binding sites relative to the center of bidirectional piRNA clusters. (*E*) Length profile of sense/antisense Tu-SINE2/3- and LINE1-related piRNAs.

In previous studies it was shown, that piRNAs bound to different Piwi paralogs exhibit different length profiles combined with a different prevalence of sense- and antisense-TE sequences (Aravin et al. 2006; Girard et al. 2006; Brennecke et al. 2007). We checked whether different Piwi paralogs left according footprints in our data set. Considering the entirety of TE-related piRNAs we did not observe different length profiles comparing sense- and antisense-TE sequences. However, when focusing on specific TE classes we detected differences in length for LINE1 related sense- (peak at 30 nt) and antisense-piRNAs (peak at 29 nt). Different length profiles are also apparent in the 23/24 nt range. Sense-piRNAs related to Tu-SINEs2/3 and LINE1 elements display a local maximum at 23 nt followed by a local minimum at 24 nt. The according antisense-piRNAs show an inversed tendency (Fig. 2E). Together, this may point to the presence of different piRNA-subpopulations presumably bound to different Piwi proteins.

### Prediction and characterization of piRNA clusters

A large fraction of piRNAs maps to one of 47 predicted piRNA clusters ranging from ∼5 to 80 kb encoding 85–6062 nonidentical piRNAs (Supplemental Table 2). Most clusters exhibit a monodirectional topology with the majority of mapped sequences distributed on one strand. Seven clusters display a bidirectional topology (Fig. 2A). We assume that these clusters share a centrally located promoter from which transcription starts on both strands in opposite directions. In order to identify factors that are possibly involved in the transcriptional regulation of piRNA clusters we searched for putative binding sites of testis-expressed transcription factors within piRNA clusters. In line with previous findings which show that A-Myb binds to pachytene piRNA promoters in mouse (Li et al. 2013) we identified 30 A-Myb binding motifs within the predicted piRNA clusters. In addition, we found a surprisingly high number (*n* = 119) of RFX4 binding motifs. Interestingly, RFX4 is highly expressed in testis and possibly interacts with RFX2 which is supposed to act as a downstream amplifier of A-Myb in mouse spermatogenesis (Wolfe et al. 2008; Horvarth et al. 2009). Therefore, we consider it possible that this downstream amplification is linked to RFX4 binding to piRNA clusters. In order to check whether the observed number of A-Myb and RFX4 binding motifs significantly exceeds the expected value we compared the number of detected binding motifs in piRNA clusters with their total genomic frequency. Indeed, we observed a significant enrichment of RFX4 (*P* = 0.026) as well as A-Myb (*P* < 0.001) binding motifs within piRNA clusters pointing to their functional importance in the transcriptional control of piRNA clusters. Since RFX4 was yet not considered to be involved in piRNA biogenesis we tested for an evolutionary conserved role of RFX4 in the transcriptional regulation of piRNA clusters in mammals and performed the same analyses on available mouse and human data sets. Notably, mouse piRNA clusters also exhibit a significant enrichment of both A-Myb (1.99-fold) and RFX4 (1.31-fold) binding motifs (*P* < 0.001) while human piRNA clusters only show a significant (*P* < 0.001) enrichment for A-Myb motifs (1.67-fold) and a marginal enrichment for RFX4 motifs (1.04-fold) that fails to reach significance (*P* = 0.45). In tupaia, A-Myb binding sites are often but not exclusively linked to the center of bidirectional piRNA clusters while RFX4 binding sites display a more dispersed distribution with only weak association to the center of bidirectional piRNA clusters (Fig. 2D).

In line with findings from previous studies on mammalian piRNAs, most cluster-encoded piRNAs map to unique loci of the genome and only 16.1% of tupaia piRNAs map to TEs (Supplemental Table 3). Consequently, when analyzing the content of TE-related sequence in piRNA clusters we found that piRNA clusters are depleted in TEs with respect to the overall genomic TE content (Fig. 2B; Supplemental Table 4). Yet the different classes of TEs are represented in piRNA clusters roughly according to their genomic frequency with TUS and Tu-III elements accounting for the majority of TEs. The overall ratio of sense and antisense piRNAs is nearly equal (3309/3878 normalized hits). However, regarding piRNAs matching to Tu-SINEs2/3 and LINE1 elements we observed a pronounced enrichment of antisense piRNAs (648/913 and 335/928 normalized hits, respectively). In comparison, piRNAs mapped to Tu-SINEs2/3 and LINE1 copies outside of piRNA clusters are biased toward sense sequences (2353/1419 and 517/317 normalized hits, respectively, Fig. 2C). This suggests that while being depleted in overall TE content, primary piRNA cluster transcripts and the resulting mature piRNAs are biased toward antisense-TE sequences that allow Piwi proteins to target active TE copies located outside of piRNA clusters.

### piRNA clusters in mammalian evolution

Drastic genomic changes including the burst of Alu elements occurred on the primate lineage after tupaias split off during Euarchontan divergence. Lineage-specific TEs like B1 or Tu-III contributed in shaping the genomes of mouse and tupaia, respectively. Phylogenetic analyses point to a very short time span between the consecutive split-off events leading to extant Glires and Scandentia suggesting similar sequence divergence rates in pairwise comparisons of human, tupaia, and mouse (Song et al. 2012; Morgan et al. 2013). However, the mouse/rat lineage apparently evolved more rapidly which should result in higher sequence divergence while making tupaia and human to appear more similar than coalescence times would suggest. To test whether the phylogenetic signals are overridden by a continuous recruiting of lineage-specific TEs into genomic piRNA clusters, we compared human, mouse, and tupaia piRNA cluster orthologs.

**FIGURE 3.**
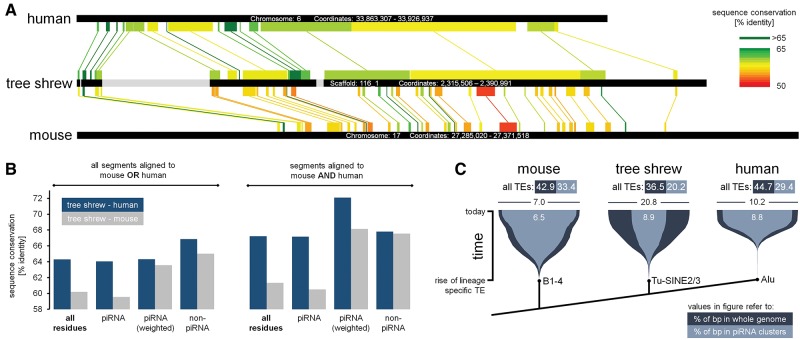
Conservation of piRNA clusters in tupaia, mouse, and human. (*A*) Conserved piRNA cluster. Aligned blocks are indicated. Gray blocks in tupaia indicate probably artificial gaps filled with N's in the tupaia genome assembly. (*B*) Different degrees of sequence conservation for different residue classes within piRNA clusters. Values are presented for all pairwise piRNA cluster alignments (*left*) and those segments of tupaia piRNA clusters that could be aligned to both mouse and human (*right*). (*C*) Accumulation of selected lineage-specific TEs in genomes (dark blue) and piRNA clusters (light blue) over time. For information, the total TE content for genomes and piRNA clusters is indicated for each species.

Initially we found that 16 and 19 of the 47 tupaia piRNA clusters are conserved in human and mouse, respectively (Fig. 3A; Supplemental Table 2). The conserved piRNA clusters comprise the largest clusters in terms of overall base pairs and encoded piRNAs and account for 70.7% and 63.6% of sequence reads mapped to tupaia piRNA clusters, respectively. We produced pairwise alignments of homologous piRNA clusters using fasta36, mapped piRNA sequences from tupaia and mouse (Peng et al. 2012) or human (Ha et al. 2014) to the obtained alignments and compared the sequence conservation of piRNA-coding residues and non-piRNA-coding residues with the overall sequence conservation.

First we noted that loci that encode piRNAs have a higher probability to produce significant alignments. In total, 50.6% of residues within the 47 tupaia piRNA clusters encode piRNAs but this number changes to 54.3% and 57.5% in tupaia–mouse and tupaia–human alignments, respectively.

The comparison of tupaia and mouse piRNA clusters revealed an overall sequence conservation of 60.2% for the aligned blocks with a total alignment size of 99,881 bp. Next we focused on piRNA-coding residues. Considering the quasi-random processing of primary piRNA cluster transcripts we assume that most residues within piRNA clusters are in principle piRNA coding. Indeed we found that 88,452 residues are represented in one (40,029 bp) or both (48,423 bp) piRNA pools and observed no difference in sequence conservation. Thus we weighted the residues according to the number of mapped sequence reads. In doing so we obtained a significantly (*P* < 0.001) increased sequence conservation of 63.6% suggesting that residues that are overrepresented in piRNAs show signs of evolutionary conservation. Surprisingly, residues that do not encode piRNAs in both species (11,429 bp) also show significantly (*P* < 0.001) increased sequence conservation (65.0%) indicating the presence of conserved and potentially regulatory elements within piRNA clusters that do not yield piRNAs (Fig. 3B). Since we could observe enrichment for RFX4 and A-Myb binding motifs we analyzed sequence conservation of these motifs and found that they exhibit increased sequence conservation with 64.1% for RFX4 and 77.8% for A-Myb motifs. As these motifs represent very few residues (295 and 90 bp, respectively) the differences in sequence conservation reaches the significance level only for A-Myb (*P* = 0.19 and *P* < 0.001, respectively).

For the tupaia–human comparison we obtained an overall sequence conservation of 64.3% (395,705 bp). Residues that encode piRNAs in one (154,600 bp) or both (205,660 bp) species do not exhibit significantly increased sequence conservation even when weighting them according to the number of mapped sequence reads. Only if we focused on residues coding for piRNAs in both species we were able to detect a slightly but still significantly higher sequence conservation of 64.9% (*P* < 0.001). As seen in the mouse comparison, residues that do not encode piRNAs (35,445 bp) do also show increased sequence conservation (66.9%, *P* < 0.001) The sequence conservation of RFX4 and A-Myb binding motifs exceeds the overall sequence conservation with 68.3% (719 bp) for RFX4 motifs and 65.5% (194 bp) for A-Myb motifs but reaches significance level only for RFX4 motifs (*P* = 0.026 and *P* = 0.71, respectively).

To elucidate whether tupaia piRNAs and piRNA clusters share more sequence similarity with mouse or with human we considered only those fragments of tupaia piRNA clusters that could be aligned to homologous mouse as well as to homologous human clusters. The overall sequence similarity compared with human amounts to 67.2% and 61.3% compared with mouse. The conservation of residues that encode piRNAs in one or both species of the pairwise comparison amounts to 72.1% (*P* < 0.001) for the tupaia–human comparison and 68.1% (*P* < 0.001) for the tupaia–mouse comparison (residues weighted according to the number of mapped sequence reads). Thus, the close phylogenetic relationship of tupaia and human in combination with the fast evolving mouse/rat lineage is reflected in a higher level of sequence similarity of both piRNA clusters and piRNAs as compared to mouse.

Relating to the role of piRNA clusters as so called transposon traps (Girard and Hannon 2008) we wanted to comprehend the spread of lineage-specific TEs regarding their total genomic abundance and their representation in piRNA clusters along time. We focused on Tu-SINE2/3 elements in tupaia, B1-4 elements in mouse and Alu elements in human since they represent the most abundant and active lineage-specific elements in these taxa. In line with the fact that mammalian piRNA clusters are poor in TE related sequences, B1-4 elements, Alu elements and in particular Tu-SINE2/3 elements are underrepresented in piRNA clusters compared with their total genomic abundance (Fig. 3C). However, their accumulation in piRNA clusters over time as deduced from the divergence from their consensus sequence coincides with the total genomic propagation progress suggesting a slowed but constantly progressing accumulation rather than distinct invasion events. Notably, by far the most pronounced depletion for TE related sequences can be found in tupaia piRNA clusters followed by human and mouse. This pattern does not change taking all TEs into account making tupaia piRNA clusters represent the TE-poorest mammalian piRNA clusters according to the current state of knowledge.

### Identification of TE derived endo-siRNAs

**FIGURE 4.**
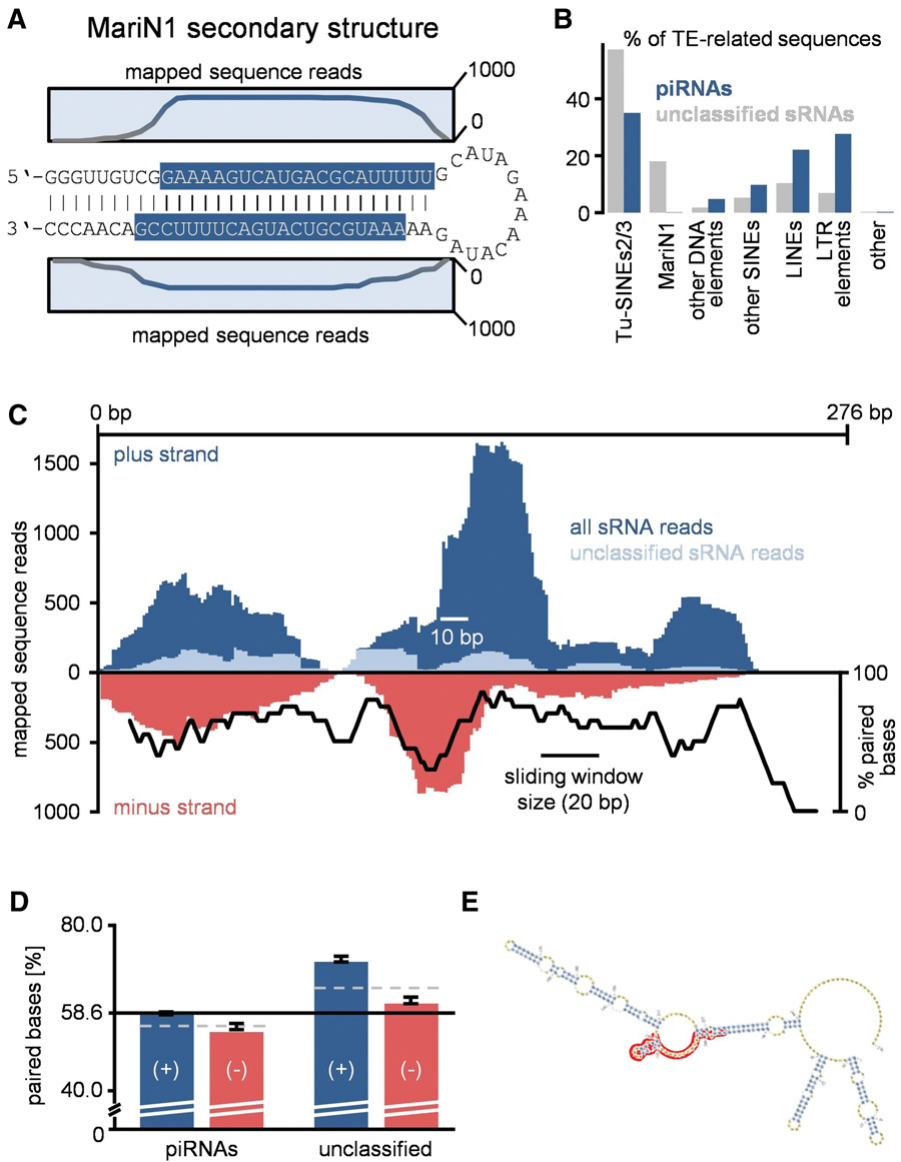
Identification of sRNA source/target sites. (*A*) MariN1 transcripts form double-stranded siRNA precursor molecules that are most likely processed by Dicer resulting in typical duplexes with 2 nt 3′ overhangs. (*B*) Sequence composition of piRNAs and unclassified RNAs with respect to different TE classes. (*C*) Distribution of mapped sense and antisense sRNA reads along the TUS consensus sequence. Sequences were mapped allowing up to three mismatches. Black chart: amount of predicted paired bases in a 20 bp sliding window. (*D*) Predicted amount of paired bases at loci targeted by piRNAs and other sRNAs. 58.6% represent the average amount of predicted paired bases for all analyzed transposon transcripts. Dashed lines indicate the average amount of paired bases for sense and antisense targeted sites. Black lines indicate the standard deviation from the average value of 100 bootstrap pseudoreplicate-data sets. (*E*) TUS secondary structure (mfold, Zuker et al. 2003) visualized with PSEUDOVIEWER2 (Han and Byun 2003). Antisense-piRNA target site is highlighted in red.

Next, we focused on sequences that neither produced matches to annotated ncRNA nor showed piRNA characteristics and checked for typical siRNA features. The biogenesis of siRNAs involves Dicer mediated cleavage of dsRNA precursor molecules that produces duplexes of typically 20–24 nt RNAs with 2 nt 3′ overhangs. This in turn results in overlaps of 18–22 bp. Although the overall amount of overlapping sequences is low, it typically amounts to 18–22 bp without displaying a peak at 10 bp (Fig. 1F), indicating the presence of siRNAs. In order to substantiate this speculation we searched for candidate source loci that can form double-stranded transcripts. We found that a large proportion of unclassified sRNAs but not piRNAs maps to the MariN1 element (Fig. 4A; Supplemental Table 4), which is a tupaia-specific high copy number MITE (miniature inverted-repeat transposable element) comprising the terminal inverted repeats from the Mariner_Tbel DNA transposon (Jurka 2009). Thus, when transcribed to RNA it forms a nearly perfect hairpin-like structure. Mapping unclassified sRNAs to the MariN1 consensus sequence revealed that all sequence reads can be exclusively assigned to the self-complementary strands mainly displaying 2 nt overhangs (Fig. 4A) while no sequences span the loop region. This strongly suggests that foldback MariN1 transcripts represent siRNA precursor molecules that are processed by Dicer resulting in mature siRNAs.

### Secondary structure of TE-transcripts correlates with sRNA target sites

Since we could not pinpoint further source loci yielding obvious dsRNA transcripts, the classification of the remaining sequences from the 18–25 nt fraction (in the following referred to as unclassified sRNAs) remained obscure. Nonetheless the unclassified sRNAs display a very similar overall amount of TE-related sequences as compared to piRNAs (15.8% and 16.1%, respectively, Fig. 4B; Supplemental Table 3) and we considered the question whether these fraction comprises further siRNAs putatively involved in TE silencing. Assuming a Dicer dependent biogenesis we would expect the unclassified sRNAs to originate from loci that yield dsRNA transcripts based on self-complementarity, thus representing adequate Dicer substrates. In contrast, we would expect that piRNAs rather tend to target sites that remain accessible due to not forming foldback secondary structures and in effect allow ping-pong mediated amplification.

Treating each of the ∼4.5 million genomic TE loci as an active element, we performed in silico RNA folding of their putative sense transcripts and compared the predicted secondary structure with the coverage of piRNAs and unclassified sRNAs. Altogether, TE sites covered by unclassified sRNAs show a higher amount of predicted base-pairing as compared with sites targeted by piRNAs (Fig. 4D). In order to obtain a refined picture, we considered the different mechanisms of siRNA and piRNA biogenesis, where a pair of siRNAs is supposed to originate from one foldback source transcript, while primary (antisense) and secondary (sense) piRNAs originate from different transcripts, namely large piRNA-cluster transcripts and TE transcripts, respectively. To this end we turned special attention on unclassified sRNAs mapped in sense orientation (putative siRNAs) and in addition on piRNAs mapped in antisense orientation as these initialize the ping-pong amplification loop. Notably, when compared with the predicted total amount of paired bases in transposon transcripts (58.6%), antisense piRNAs prefer sites with reduced base-pairing (54.8% paired bases) while putative siRNAs map to sites with increased base-pairing (71.7% paired bases). In contrast, sites covered by sense piRNAs and antisense unclassified sRNAs do not show a pronounced preference for double-stranded or single-stranded target sites (58.6% and 61.6% paired bases, respectively, Fig. 4D).

As an example, we focused on TUS elements which represent one of the most abundant TEs in the tupaia genome in terms of copy number and overall base pair counts. Furthermore TUS elements on average exhibit 98% identity to their consensus sequence suggesting this element to be still active. We observed a pronounced ping-pong signature approximately in the center of the TUS element (∼105–160 bp, Fig. 4C). Strikingly, the mapped antisense piRNAs perfectly coincide with the site that exhibits the lowest amount of paired bases except for the poly(A) tail. On the other hand the distribution of unclassified sRNAs correlates well with the amount of paired bases (Fig. 4C,E) emphasizing our results applying the genome-wide approach.

In order to test the general validity of our results we applied the same procedure to mouse and human piRNAs mapped to mouse and human TEs, respectively. Again, we found that sites targeted by antisense piRNAs exhibit significantly (*P* < 0.001) reduced amount of paired bases (52.9% and 54.1%, respectively) compared to sites corresponding to sense piRNAs (56.9% and 57.5%, respectively).

## DISCUSSION

Piwi proteins and piRNAs silence TEs in the germline of a variety of animals. Enigmatically, experiments on mouse and rat suggest that in mammals Piwi proteins and piRNAs are expressed mainly in the male germline. This is in line with the fact that male mice Piwi knockouts show increased transposon activity paired with sterility while females do not (Deng and Lin 2002; Kuramochi-Miyagawa et al. 2004; Carmell et al. 2007). On the other hand endo-siRNAs were observed to act in mouse oocytes (Tam et al. 2008; Watanabe et al. 2008), promoting the assumption that the siRNA-pathway took over piRNA-pathway function in the mammalian female germline. However, the lack of one of the four mammalian Piwi-paralogs (Piwil3) and recent findings that report on a highly active isoform of Dicer that acts in mouse and rat oocytes but not in oocytes of other rodents (Flemr et al. 2013) underline the special status of this phylogenetic lineage. In this context, the tupaia with its full set of mammalian Piwi paralogs presumably provides the more relevant model for research on mammalian RNAi. Especially regarding its close relationship to primates, insights from the tupaia may more meaningfully represent the human outgroup situation as compared to mice.

Noteworthy, our results and interpretations rest on a very limited and exclusive tissue sample from one individual. The nonavailability of replicate data sets do not allow us to asses or correct for inter-individual variability that might exist in natural tupaia populations. Yet, considering the qualitative character of our results that are scarcely affected by intraspecific quantitative variations we are convinced that our findings and interpretations are not affected.

### Length profiles of tupaia piRNAs point to four Piwi paralogs involved in piRNA biogenesis

piRNAs bound to different Piwi proteins such as Miwi, Mili, and Miwi2 in mouse or Piwi, Aub, and Ago3 in *Drosophila* each have distinct length profiles and preferentially associate with either sense or antisense piRNAs (Siomi et al. 2011). According to this, the analysis of LINE1-related sense and antisense piRNAs revealed different length profiles with sense piRNAs displaying a maximum at 30 nt and antisense piRNAs displaying a maximum at 29 nt, indicating that these piRNAs represent different piRNA populations bound to distinct Piwi proteins. However, Tu-SINE2/3-related sense and antisense piRNAs both peak at 29 nt suggesting a more complex mechanism in that not only sense/antisense-orientation but also the class of the TE transcript plays a role in the process of allocating a piRNA to a Piwi protein.

Beside the large fraction of 29/30 nt piRNAs, we found that tupaia piRNA clusters encode a considerable amount of surprisingly small-sized piRNAs (18–25 nt) below the typical mammalian piRNA size range with sense piRNAs displaying a local maximum at 23 nt and a local minimum at 24 nt while the situation for antisense piRNAs tends to be inverse. One explanation for the presence of these small-sized piRNAs could be a more progressive 3′ end trimming during piRNA maturation. That would be in line with the fact that ∼63% of the small-sized piRNAs have identical 5′ ends when compared with canonical piRNAs (Supplemental Fig. 1A). Assuming these small piRNAs to represent a sub-fraction of 29/30 nt piRNAs we would expect that 23 nt piRNAs correspond to 29 nt piRNAs while 24 nt piRNAs should correspond to 30 nt piRNAs. However, the observed length profiles for, e.g., sense and antisense LINE1-related piRNAs cannot be explained by a simple ±1 nt shift (sense = 23/30 nt versus antisense = 24/29 nt). Thus, we cannot rule out the possibility that the small-sized piRNAs represent two separate piRNA subpopulations distinct from the 29 nt and 30 nt piRNAs. The presence of four different piRNA subpopulations that show distinguishable length profiles would also be in line with the fact that the tupaia genome encodes four functional Piwi proteins and that all four Piwi paralogs are expressed simultaneously on the mRNA level in adult testis of the primate representatives human, rhesus, monkey (*Macaca mulatta*) and common marmoset (*Callithrix jacchus*, Supplemental Fig. 1B).

Co-IP experiments using Piwi paralog-specific antibodies—not necessarily in the tupaia—could throw further light upon mammalian Piwi/piRNA systems that comprise the standard set of four Piwi paralogs.

### Different conservation patterns within piRNA clusters

Our results reveal that piRNAs are largely produced from orthologous loci in euarchontogliran mammals. Though these piRNA clusters are considerably less conserved as compared with protein coding genes on the DNA level, we showed that piRNA-coding residues that are overrepresented in piRNA pools exhibit elevated sequence conservation as was previously described when comparing piRNA clusters in mouse and rat (Lau et al. 2006). Interestingly, loci that do not encode piRNAs but may have regulatory functions such as A-Myb and RFX4 binding motifs show an even higher level of sequence conservation which gives the impression that the ability of a certain genomic locus to produce piRNAs is more important than preserving primary sequences of piRNAs over evolutionary time scales.

Apparently mammalian piRNA clusters are prevented from being invaded by TEs which at first sight is in contrast to the idea initially formulated for invertebrate model taxa in that piRNA clusters represent transposon traps. However, it has been described previously that mammalian pachytene piRNA clusters are rather TE poor (Beyret et al. 2012; Nordstrand et al. 2012). Thus, we initially hypothesized that once a new TE entered a piRNA cluster allowing the biogenesis of anti-TE piRNAs, there could be either a selective constraint or the emergence of a sequence evolution determining mechanism both acting against the further accumulation of these elements in piRNA clusters. As a consequence we would expect that the divergence of TEs from their consensus sequence is higher in piRNA clusters than in the whole genome indicating more ancient insertions. However, the observed divergence rates of TEs inside and outside of piRNA clusters did not support this hypothesis but rather point to a constantly reduced insertion rate. We speculate that active TEs inside transcriptionally active regions represent a threat that might be the trigger to develop insertion avoidance mechanisms and/or selective regimes over evolutionary time scales that prevent the accumulation of TEs in piRNA clusters and are responsible for the low TE content observed in piRNA clusters. Interestingly, tupaia piRNA clusters display the strongest bias toward non-TE sequences. This suggests that they are either most sensitive for disruptive TE insertions resulting in strong negative selection or alternatively are sheltered in some way and to some degree, possibly due to a tighter regulation of their chromatin state.

### Different target sites for endo-siRNAs and piRNAs in the tupaia male germline

Our analyses show that tupaia antisense piRNAs arise from large genomic piRNA clusters and preferentially map to TE transcripts at sites that represent adequate targets for single-stranded guiding RNAs due to reduced base-pairing. Furthermore, we describe siRNAs that originate from sites that tend to form foldback secondary structures and thus are not accessible for piRNAs. This is supported by the fact, that hairpin-like MariN1 transcripts are virtually exclusively covered by siRNAs although MariN1 copies are present in piRNA clusters. Hence, secondary structure of MariN1 transcripts seems to efficiently prevent piRNA targeting and ping-pong amplification which is in agreement with previous findings that target secondary structure is a major determinant of RNAi efficiency (Shao et al. 2007). Since MariN1 transcripts are hardly accessible for any type of guiding RNAs, whether siRNAs or piRNAs, Dicer dependent processing of TE transcripts itself would represent the crucial silencing step. Thus, we suppose that Dicer in combination with piRNA-guided Piwi proteins form a powerful defense line that cannot be bypassed by foldback TE transcripts that avoid recognition by single-stranded guiding RNAs. It is worth mentioning that this by no means rules out the possibility that, e.g., MariN1 derived siRNAs subsequently guide effector proteins to full-length Mariner_Tbel transcripts or other targets, resulting in transcriptional regulation or post-transcriptional cleavage.

Reviewing previous studies on *Drosophila* sRNAs we hypothesize that our conclusions drawn on the basis of the tupaia data could also be valid for sRNA silencing pathways in *Drosophila*. In *Drosophila*, siRNAs were assumed to silence TEs mainly in the soma, displaying a more or less separation of the siRNA- and piRNA-pathway into soma and germline (Ghildiyal et al. 2008). However, TE-derived si- as well as piRNAs were sequenced from the *Drosophila* ovary somatic sheet cell line (Lau et al. 2009). In addition, Lau and coworkers observed differences in the siRNA/piRNA ratio for different TEs, as we did in tupaia, but did not speculate about possible causes. We suppose that secondary structure of target transcripts should generally affect sRNA silencing pathways in any species. At a first glance, analyses of both mouse and human piRNAs confirmed this assumption in that antisense piRNAs map to transposon sites with reduced base-pairing compared with sense piRNAs. Therefore, it would be surely worthwhile to conduct an in depth examination and check for a possible coherence of TE transcript secondary structure and the siRNA/piRNA ratio in additional species.

### A-Myb and RFX4 are candidates for the transcriptional regulation of tupaia piRNA clusters

The role of A-Myb as a transcription factor regulating piRNA cluster transcription appears to be conserved in mammals as we identified putative A-Myb binding sites linked to the center of bidirectional tupaia piRNA clusters. In addition, the ubiquity of putative RFX4 binding sites in tupaia and mouse piRNA clusters suggests that RFX4 also plays a role in the transcriptional regulation of piRNA clusters during mammalian spermatogenesis. This idea is supported by the observation, that RFX4 motifs exhibit increased sequence conservation in pairwise sequence comparisons with both mouse and human. Interestingly, despite increased sequence conservation RFX4 binding motifs are not significantly enriched in human piRNA clusters which is presumably caused by loss of RFX4 binding sites in piRNA clusters on the lineage leading to human.

RFX transcription factors bind to the testis-specific histone H1t promoter (Wolfe et al. 2008), however, they were not linked to piRNA biogenesis up to now. As RFX factors are supposed to act downstream from A-Myb (Horvath et al. 2009) we speculate that RFX4 may amplify piRNA cluster transcription in a A-Myb-dependent manner. Considering the distribution of putative RFX4 binding sites that show only weak association to the centers of bidirectional piRNA clusters, we assume that RFX4 transcription factors may generally act as a transcriptional enhancer. Since both A-Myb and RFX4 binding sites are also present in monodirectional piRNA clusters, RFX4 putatively affects which strand is dominant. Further analyses including ChIP-sequencing using antibodies specific for RFX4 are needed to elucidate the functional role of RFX4 in piRNA biogenesis.

In summary, our data show that siRNA- and piRNA-pathways in mammals are not generally allocated to the female and male germline, respectively, but rather functionally complement each other. This suggests that small RNA-based TE defense can take advantage of the different abilities of both piRNA- and siRNA-pathway components to form an effective protection against mobile DNA. Thus, TE defense that involves components of the siRNA-pathway may play a more important role in animal's male or female germlines than commonly thought. Considering the plasticity of TE repertoires over evolutionary timescales, we expect future studies on additional mammals to reveal further interesting insights into small RNA mediated TE defense.

## MATERIALS AND METHODS

### Extraction of small RNAs (sRNAs)

Total RNA was isolated from testis tissue of *Tupaia belangeri* with TRI Reagent (Sigma-Aldrich) and run on a 12% urea-based denaturing acrylamide gel together with GeneRuler Ultra Low Range DNA Ladder for 30 min at 1200 V and 50 mA. The gel was stained for 2 min using ethidium bromide/H_2_O solution (0.5 µg/mL) and subsequently destained for 2 min in nuclease free water. The sRNA fraction corresponding to a size of ∼20–35 bp was excised from the gel and dissolved in H_2_O with the Ultrafree-MC system (Millipore). The RNA eluate was concentrated to a total volume of 50 µL and desalted using the Amicon Ultra 3K system (Millipore).

### Periodate treatment and β elimination

In order to enrich our sRNA library for piRNA sequences we performed periodate treatment and β elimination according to the method applied by Rajasethupathy et al. (2012) with minor adjustments with respect to the applied RNA sample volume. Periodate treatment and β elimination was performed in parallel using synthetic 3′-2′-O-Met modified and unmodified RNA oligonucleotides as controls. Although the controls indicated an efficient degradation of nonmethylated RNAs our subsequent results suggest that a fraction of putative siRNAs was resistant to periodate treatment. However, since we used a severely limited and unique tissue sample we could not carry out a comparative technical approach with preparing and sequencing both periodate-treated and untreated libraries to reliably test periodate treatment efficiency and methylation status of the sequenced RNAs.

### sRNA library preparation

A 5′-activated and 3′-blocked RNA adapter (5′-AppCUGUAGGCACCAUCAAUddC-3′) was ligated to the 3′ end of periodate-resistant RNAs in the absence of ATP. The ligation product was purified via ethanol precipitation and separated from unligated RNA and adapter molecules by denaturing PAGE. The ligation product was excised from the gel, dissolved, concentrated, and desalted as described above using an Amicon Ultra 10K filter device. Following the ligation of a second RNA adapter (5′-GACUGGAGCACGAGGACACUGACAUGGACUGAAGGAGUAGAAA-3′) to the 5′ end of the RNA in the presence of ATP, RNA was purified via ethanol precipitation, reverse-transcribed using Invitrogen's Superscript III (primer for first strand synthesis: 5′-CTGTAGGCACCATCAAT-3′) and PCR amplified (forward primer: 5′-ACATGGACTGAAGGAGTAGA-3′, reverse primer: 5′-CTGTAGGCACCATCAAT-3′). The PCR was carried out according to the following thermal cycling profile: 3′ at 95°C, [30′′ at 95°C, 30′′ at 51°C, 20′′ at 74°C] × 30, 5′ at 74°C. Sequencing of the obtained library was performed on an Illumina HiSeq 2000 system.

### Piwil1-4 expression analysis

We used total testis RNA extracts from *Homo sapiens* (AMS Biotechnology), *Macaca mulatta*, and *Callithrix jacchus* to synthesize cDNA with Invitrogen's Superscript III and perform PCR with Piwi paralog specific and exon-spanning primers that were designed using the easyPAC software (Rosenkranz 2012). For each species and Piwi paralog we performed PCRs applying different numbers of PCR cycles (25, 30, 35, and 40) and visualized PCR products on an ethidium bromide-stained agarose gel using SpeedyQuant pro (in-house software, available upon request). The PCR products were Sanger sequenced in order to verify that the PCR products correspond to spliced Piwi mRNAs.

### Sequence annotation

sRNA sequences were mapped to the *Tupaia belangeri chinensis* genome (Fan et al. 2013) using SeqMap (Jiang and Wong 2008) allowing only perfect matches. We screened successfully mapped sequences against available tupaia noncoding RNA sequences (Ensembl database release 73), human miRNA sequences (Griffiths-Jones 2004), as well as tRNA and rRNA sequences downloaded from RepBase (Jurka et al. 2005).

A complete set of annotated ancestral and lineage-specific tupaia transposon sequences was downloaded from RepBase (Jurka et al. 2005) and mapped to the genome of *Tupaia belangeri chinensis* using the RepeatMasker software version 4.0.2 (AFA Smit, R Hubley, P Green, unpubl.) applying the most sensitive settings (slow search, search engine: cross_match). In order to identify the amount and composition of transposon related sRNAs, the obtained repeat annotation was compared with the coordinates of mapped sRNAs. The same procedure was applied using the *Tupaia belangeri* gene annotation from Ensembl database. Values were normalized by the number of genomic hits produced by each sequence. Numbers related to sRNA TE annotation in Figures 1 and 2 refer to nonidentical sequences. According analyses using read counts yielded essentially identical results. Numbers related to coverage of TEs in Figure 4 refer to sequence read counts which is more meaningful in this context. The length distribution, base composition and 5′ overlap of mapped sequences was analyzed using in-house Perl scripts that are available upon request.

### piRNA cluster prediction

Sequences that were not annotated on the basis of known ncRNA were used to predict piRNA clusters with proTRAC version 2.0.2 (Rosenkranz and Zischler 2012) that is available at http://sourceforge.net/projects/protrac/. Sequence motifs of MYB-A and RFX4 transcription factors were obtained from UniPROBE database (Newburger and Bulyk 2009). The piRNA clusters were predicted using the default settings with normalization by the number of sequence reads (command line option -nr) and number of genomic hits (command line option -nh). Human and mouse piRNA clusters were predicted accordingly using piRNA sequences deposited at NCBI's sequence read archive (SRA) under the accession SRX271416 (Ha et al. 2014) and SRX154530 (Peng et al. 2012), respectively. The predicted human and mouse piRNA clusters are listed in Supplemental Tables 5 and 6. The TE content of predicted piRNA clusters was determined using the RepeatMasker software and species-specific TE libraries from RepBase (Jurka et al. 2005).

### Evolutionary analyses of piRNA clusters and piRNA sequence conservation

In order to find orthologous, syntenic piRNA producing loci across mouse, tupaia, and human we used the sequences of predicted tupaia piRNA clusters to perform a BLASTN search in the genomes of mouse and human (both genome assemblies downloaded from Ensembl database version 76.38). Blocks of contiguous alignments in correct order were assumed to represent homologous loci. The obtained coordinates were compared with the proTRAC piRNA cluster annotation for mouse and human. If homologous loci were found to represent piRNA clusters in both species the piRNA clusters were realigned using fasta36 and ssearch36 (FASTA package version 36.3.6, Pearson and Lipman 1988) to ensure the highest possible sensitivity. We decided to continue with the alignments obtained by applying fasta36 since they showed a higher sequence identity and in total covered more base pairs. We then mapped the piRNA sequences to the piRNA cluster alignments and compared the conservation of piRNA-coding residues versus the entirety of aligned residues. piRNA-coding residues were weighted by the number of mapped piRNA reads with correction of the different total number of mapped reads for the two species in question. The applied in-house Perl scripts are available upon request.

### Statistical analysis

For the conducted significance tests, χ^2^ values were calculated from 2 × 2 contingency tables. *P* values refer to the right-tailed probability of the χ^2^ distribution (degrees of freedom = 1). Contingency tables, χ^2^ values and exact *P* values for each significance test are provided in Supplemental Table 7.

### Analysis of secondary structure of sRNA target sites

The secondary structure of putative TE sense transcripts of ∼4.5 million identified TE loci in the tupaia was predicted using the command-line version of RNAfold from the ViennaRNA Package without allowing lonely pairs (Lorenz et al. 2011). The position of predicted paired and unpaired bases was compared with the coverage of perfectly mapping sRNA sequence reads using an in-house Perl script that is available upon request. The same procedure was applied to 100 pseudo replicate-data sets generated by bootstrapping of the original sequence data set resulting in data sets comprising the same quantity of sequences as the original data set. Analogous analyses were carried out using human and mouse piRNA data sets (Peng et al. 2012; Ha et al. 2014) and identified TE sequences in the human and mouse genome.

## DATA DEPOSITION

Sequence data sets and project information are available at NCBIs SRA under the BioProject ID PRJNA240268.

## SUPPLEMENTAL MATERIAL

Supplemental material is available for this article.

## Supplementary Material

Supplemental Material
