# Supplementary material for: Tupaia small RNAs provide insights into function and evolution of RNAi-based transposon defense in mammals
Source: RNA. 2015 May;21(5):911–22. doi: 10.1261/rna.048603.114 (PMC4408798; doi:10.1261/rna.048603.114)
Supplement: Supplemental Material [file supp_21_5_911__index.html]

Tupaia small RNAs provide insights into function and evolution of RNAi-based transposon defense in mammals — Tupaia small RNAs provide insights into function and evolution of RNAi-based transposon defense in mammals — Supplemental Material 

# Tupaia small RNAs provide insights into function and evolution of RNAi-based transposon defense in mammals

## Supplemental Material

**Files in this Data Supplement:**

- Supp Table 1.xlsx
- Supp Table 4.xlsx
- Supp Table 7.xlsx
- Supp Table 3.xlsx
- Supp Table 5.xlsx
- Supp Table 2.xlsx
- Supp Table 6.xlsx
- Supp Figure 1.jpg
- Supp Fig Legend.docx
