# Supplementary figures and images for: Tupaia small RNAs provide insights into function and evolution of RNAi-based transposon defense in mammals
Source: RNA. 2015 May;21(5):911–22. doi: 10.1261/rna.048603.114 (PMC4408798; doi:10.1261/rna.048603.114)

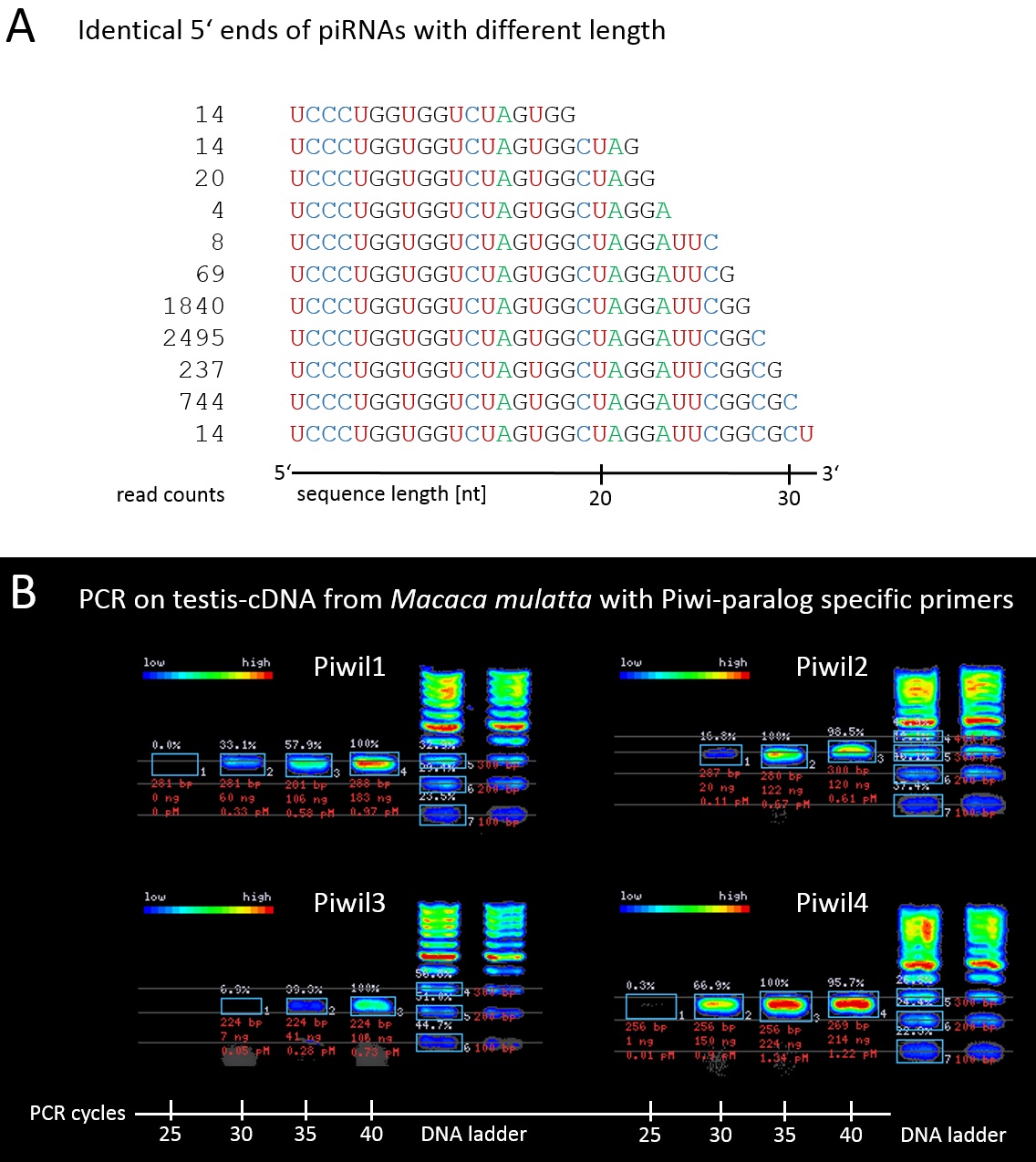

Supplement: Supplemental Material [file supp_048603.114_SuppFigure_1.jpg]
