# Supplementary material for: Tupaia small RNAs provide insights into function and evolution of RNAi-based transposon defense in mammals
Source: RNA. 2015 May;21(5):911–22. doi: 10.1261/rna.048603.114 (PMC4408798; doi:10.1261/rna.048603.114)
Supplement: Supplemental Material [file supp_048603.114_SuppFigLegend.docx]

Supplemental Figure 1. (A) piRNAs with different length exhibit identical 5’ ends. (B) Piwil1-4 are expressed on the mRNA level in *Macaca mulatta* (as well as in *Homo sapiens* and *Callithrix jacchus*, data not shown).
